# The last frontier: transcatheter devices for percutaneous or minimally invasive treatment of chronic heart failure

**DOI:** 10.1007/s12471-017-1018-8

**Published:** 2017-07-24

**Authors:** V. J. Nijenhuis, L. Sanchis, J. A. S. van der Heyden, P. Klein, B. J. W. M. Rensing, A. Latib, F. Maisano, J. M. ten Berg, P. Agostoni, M. J. Swaans

**Affiliations:** 1 Department of Cardiology, St. Antonius Hospital, Nieuwegein, The Netherlands; 2 Cardiovascular Institute, Hospital Clinic, Barcelona, Spain; 3 Department of Cardio-Thoracic Surgery, St. Antonius Hospital, Nieuwegein, The Netherlands; 4 Interventional Cardiology Unit, San Raffaele Scientific Institute, Milan, Italy; 5 Interventional Cardiology Unit, EMO-GVM Centro Cuore Columbus, Milan, Italy; 6 University Heart Centre, University Hospital Zurich, Zurich, Switzerland

**Keywords:** Heart failure, Transcatheter, Minimally invasive, Ventricular aneurysm, Tricuspid regurgitation, Neuromodulation

## Abstract

Heart failure has a high prevalence in the general population. Morbidity and mortality of heart failure patients remain high, despite improvements in drug therapy, implantable cardioverter-defibrillators and cardiac resynchronisation therapy. New transcatheter implantable devices have been developed to improve the treatment of heart failure. There has been a rapid development of minimally invasive or transcatheter devices used in the treatment of heart failure associated with aortic and mitral valve disease and these devices are being incorporated into routine clinical practice at a fast rate. Several other new transcatheter structural heart interventions for chronic heart failure aimed at a variety of pathophysiologic approaches are currently being developed. In this review, we focus on devices used in the treatment of chronic heart failure by means of left ventricular remodelling, left atrial pressure reduction, tricuspid regurgitation reduction and neuromodulation. The clinical evaluations of these devices are early-stage evaluations of initial feasibility and safety studies and additional clinical evidence needs to be gathered in appropriately designed clinical trials.

Heart failure (HF) is a major public health issue in both developed and developing countries [1]. In the past 2 decades, developments in drug therapy, implantable cardioverter-defibrillators and cardiac resynchronisation therapy, as well as mechanical circulatory support with left ventricular assist devices and heart transplantation have improved the prognosis of patients with HF and a reduced ejection fraction (HFrEF) [1]. However, morbidity and mortality rates remain high [2]. Furthermore, there is no effective pharmacological treatment for HF patients with a preserved ejection fraction (HFpEF) [3].

In an attempt to further improve the prognosis and quality of life of HF patients, several transcatheter implantable devices have emerged. In this review, we aim to describe a spectrum of recently introduced devices for the treatment of chronic HF by means of left ventricular (LV) remodelling, reduction of left atrial (LA) pressure, reduction of tricuspid regurgitation and neuromodulation. To provide an overview of these devices, this review focusses on describing these devices, their main procedural characteristics, patient eligibility, procedural results and clinical outcomes. Transcatheter devices for aortic valve implantation, mitral valve replacement/repair, paravalvular leak closure, percutaneously delivered biological therapies and interventions for acute HF fall beyond the scope of this review.

## Left ventricular remodelling

**Fig. 1:**
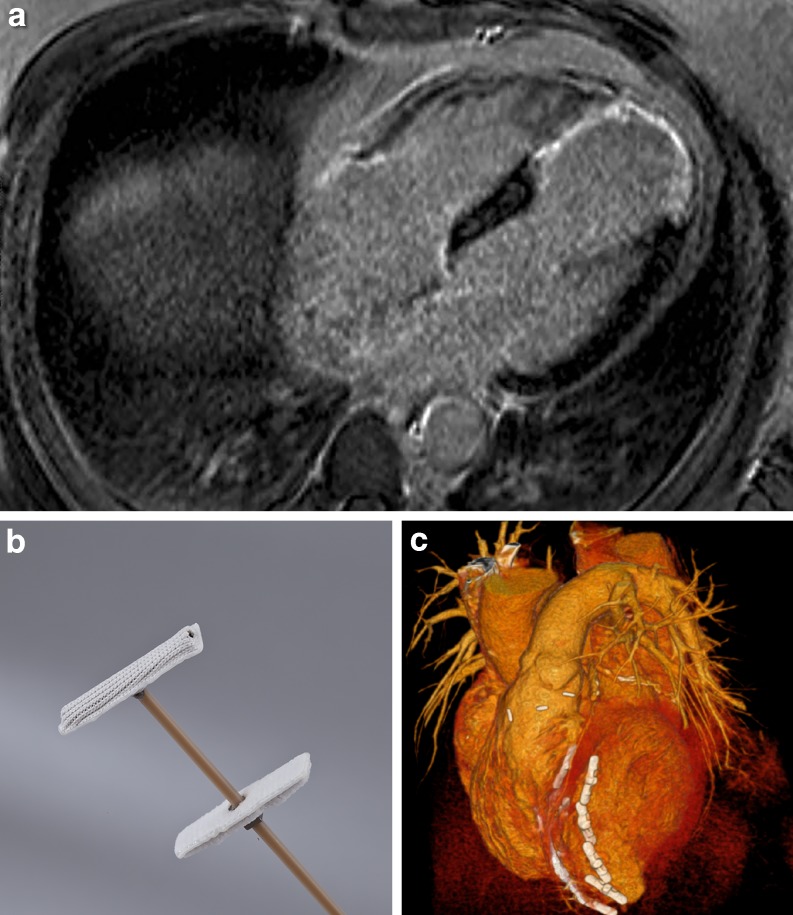
Left ventricular remodelling. **a** Transversal plane of magnetic resonance imaging showing left ventricular aneurysm caused by apical myocardial infarction, as visualised by late enhancement. **b,c** Revivent TC Ventricular Enhancement System (BioVentrix; San Ramon, CA, USA). Internal and external anchors of the device work together to exclude scarred tissue of the left ventricle (**b**). A computed tomography scan reconstruction of the heart (anterior view) after the implantation of a series of anchor pairs (**c**). (Images adapted with permission from BioVentrix; San Ramon, CA, USA)

Ischaemic heart disease is one of the main causes of HFrEF [4]. Scar formation after myocardial infarction results in progressive LV remodelling (Fig. 1a; [4]). Expansion and thinning of the left ventricle increases its radius and wall tension, leading to a loss of the typical LV cone shape [5], thereby inducing inefficient ventricular contraction and subsequent HF onset.

In classical surgical ventricular reconstruction procedures, the LV aneurysm is resected to reduce volume and obtain an elliptical shape. Results in multicentre registries for patients with ischaemic cardiomyopathy have been promising [6–11]. However, the only randomised trial thus far (STICH: Surgical Treatment for Ischemic Heart Failure) failed to demonstrate any benefit in the composite endpoint of death and rehospitalisation for cardiac causes [12]. Moreover, these procedures have a high surgical risk related to cannulation, cardiopulmonary bypass and ventriculotomy. That is why they have been largely abandoned in routine clinical practice. New, less invasive, transcatheter approaches for ventricular reconstruction have recently emerged (Tab. 1).

**Table 1:** Transcatheter options for left ventricular reconstruction

Device	Status	Indication	Method	Access	Off-pump
Revivent (TC)	CE mark December 2012 Revivent; CE mark June 2016 Revivent TC	Anteroseptal, anterior or apical scarring incl. pseudochordae and LVDD >70 mm [13]	Reshaping LV by means of plication wires and titanium anchors	Medio-sternotomy Revivent; Mini-thoracotomy and jugular Revivent TC	Yes
Parachute	CE mark October 2012	Anteroseptal, anterior or apical scarring	Expansion of ePTFE membrane on a nitinol frame configured as an umbrella to exclude the aneurysm	Percutaneous	Yes

*CE* Conformité Européene,* ePTFE* expanded polytetrafluoroethylene*, LV* left ventricle*, LVDD* left ventricular diastolic diameter

### Revivent

The Revivent TC Ventricular Enhancement System (BioVentrix; San Ramon, CA, USA) allows major LV reconstruction on a beating heart without cardiac incisions. The device is composed of titanium anchor pairs covered in polyester cloth (Fig. 1b) and connected by an adjustable-length tether made of high-strength biocompatible poly-ether-ether ketone.

First, the scarred LV segments are exposed. A dedicated curved needle is inserted in the LV free wall to reach the septum and right ventricle where its tip can be ‘captured’ through a jugular venous access catheter. This creates a connection wire between the LV access site and the jugular access site. Over this wire, a fastening anchor is introduced through the catheter and subsequently retracted until it comes into contact with the right ventricular side of the intraventricular septum. Next, the catheter is removed and another fixing anchor is placed on the outer face of the ventricular wall via the site of needle insertion. Several anchors are implanted until optimal plication of the aneurysm is achieved (Fig. 1c).

Initial, published, data showed good durability and satisfactory results of the Revivent device in 11 patients [13]. Recently, data were presented on the implantation in 71 patients treated with either the original delivery system that required a sternotomy (Revivent; 51 patients) or the Revivent TC System (20 patients). These data showed a reduction of LV volume, increase of LV ejection fraction and increase in 6‑minute walk distance [14]. To confirm results and gather data on long-term 5‑year safety, BRAVE-TC (BioVentrix Registry Assessment of Ventricular Enhancement for the Revivent TC) is currently recruiting up to 100 subjects.

### Parachute

The Parachute device (CardioKinetix; Menlo Park, CA, USA) is comprised of a self-expanding frame, a polytetrafluoroethylene impermeable membrane and an atraumatic foot. The nitinol frame has a conical shape with 16 struts that ends in a 2 mm anchor to engage the myocardium for device stabilisation. The distal foot is radio-opaque and provides a contact point between the LV apex and the device in addition to facilitating visualisation for placement. The device is available in 4 sizes, all in two heights.

Upon implantation, the left ventricle is accessed via the transfemoral approach using a conventional pig-tail wire. A stiff wire is then positioned for support and finally a pre-shaped catheter is placed near the LV apex. The device is advanced through the sheath until the foot is exposed and is advanced further until the foot contacts the apex. The device is deployed by pulling back on the guide catheter while the delivery catheter is held motionless, i. e. the device is ‘unsheathed’ by pulling back the outer sheath. The self-expansion is facilitated by inflating a low-pressure balloon until the anchors are fully expanded and in contact with the LV wall. Contrast angiography of the left ventricle is performed to confirm positioning before releasing the device.

The first-in-human study (PARACHUTE IV: PercutAneous Ventricular RestorAtion in Chronic Heart FailUre due to Ischemic HearT DiseasE), demonstrated feasibility and safety of the device in 34 patients [15]. Procedural success was 91% with a significant reduction of diastolic volume. Moreover, 85% of patients improved in their functional class. Currently, the pivotal randomised PARACHUTE V trial (NCT01614652) is recruiting to test the parachute device versus optimal medical therapy in 560 patients. The study is almost at its halfway enrolment point. Furthermore, the non-randomised observational PARACHUTE V trial (NCT02543632) is recruiting to assess quality of life and cardiac output benefit at six months in 105 patients (25 controls).

## Percutaneous tricuspid valve repair

The main causes of tricuspid regurgitation (TR) are annular dilation and right ventricular enlargement, often secondary to left-sided heart disease [16]. Moderate to severe TR significantly impacts functional status and is an independent risk factor for poor long-term survival [17–20]. In high-risk patients with an advanced state of disease, tricuspid surgery carries an operative mortality of up to 22% and is therefore frequently averted [21].

The anatomy of the tricuspid valve apparatus is complex. The aim of minimally invasive approaches for the treatment of severe, symptomatic TR is placement of transcatheter valves, either in the position of the native tricuspid valve or in the caval veins. Here, we will discuss the devices for direct transcatheter tricuspid repair (Tab. 2).

**Table 2:** Transcatheter options for transcatheter tricuspid valve repair

Device	Status	Method	Size	Access	Repositionable
4TECH TriCinch System	First-in-man CE mark multicentre trial [23]	Annulus corkscrew connected through a band to a stent in the IVC	27, 32, 37 and 43 mm (stent)	21.3 F	Yes
Forma Repair System	First-in-man [24]	Foam-filled polymer balloon spacer fixated in the RV apex	12 and 15 mm	24 F	Yes
Trialign	Compassionate use [25]; Completed FDA early feasibility study (SCOUT) Multicentre CE mark Trial (SCOUT-II)	Pairs of pledgets to plicate the posterior annulus and bicuspidize the valve	10, 14, 17, 21 mm (bident span)	14 F	–
Mitraclip	Compassionate use [26–28]	Edge-to-edge repair		24 F	Yes
Millipede annular ring	First-in-man	Adjustable direct annuloplasty ring	NA	NA	Yes
Cardioband Tricuspid	Preclinical (CE mark mitral cardioband)	Supra-annular fixation sleeve with anchors	6 sizes (A–F)	25 F	Yes

*CE* Conformité Européene*, IVC* inferior vena cava,* NA* not available*, RV* right ventricle*, NA* not applicable

### TriCinch system

**Fig. 2:**
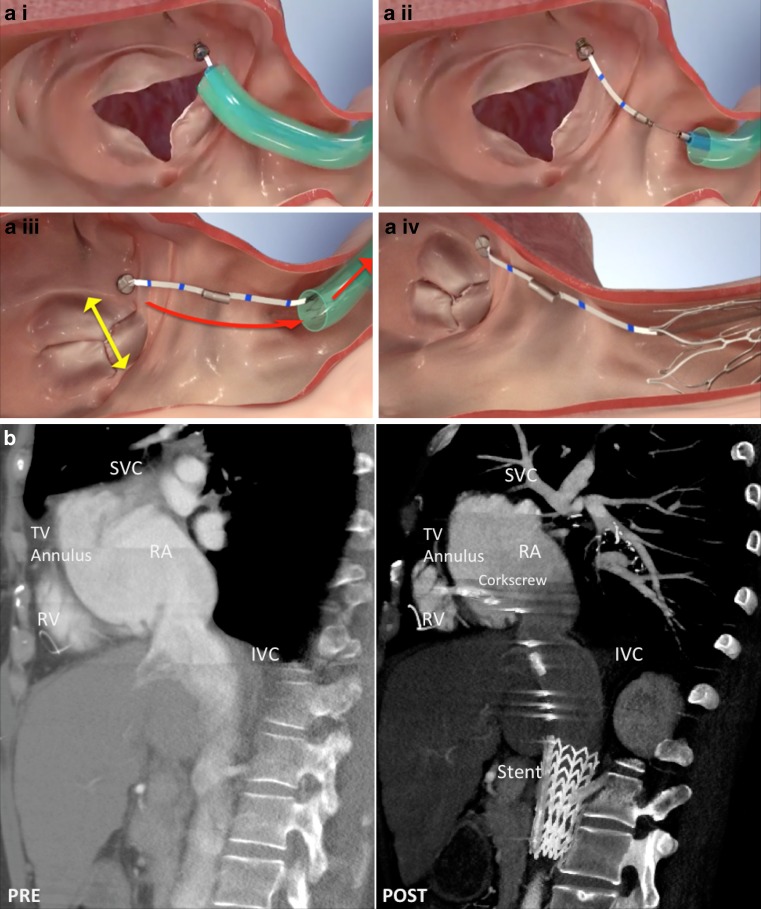
Tricuspid valve repair. Several devices are being tested for tricuspid valve repair, for example the TriCinch System (4Tech Cardio Ltd., Galway, Ireland). **a** Implantation steps of the TriCinch System. A stainless steel corkscrew is fixated into the anteroposterior tricuspid annulus *(i)* to assure the stability of the implant. By pulling the system through a Dacron band *(ii)*, the anchoring corkscrew remodels the anteroposterior annulus *(iii)*. The tension is maintained by fixation of a self-expanding nitinol stent in the inferior vena cava *(iv)*. **b** Computed tomographic sagittal images of the TriCinch System pre (*left side*) and post implantation (*right side*). (*IVC* inferior vena cava, *RA* right atrium, *RV* right ventricle, *SVC* superior vena cava, *TV* tricuspid valve. Images adapted with permission from 4Tech Cardio Ltd., Galway, Ireland)

The TriCinch System (4Tech Cardio Ltd., Galway, Ireland) is a percutaneous device designed for tricuspid valve remodelling. Transfemoral fixation of a stainless steel corkscrew into the anteroposterior tricuspid annulus is performed to assure stability. By pulling the system towards the inferior vena cava through a Dacron band the anchoring corkscrew remodels the annulus and tension is maintained by fixation of a self-expanding nitinol stent in the inferior vena cava (Fig. 2). The stent is available in multiple sizes, allowing a total indicated vessel diameter ranging from 18 to 35 mm.

The first-in-man case demonstrated the feasibility of the TriCinch device and showed a reduction in annular dimensions and TR severity [22]. The TriCinch is to be evaluated in the PREVENT trial (Percutaneous Treatment of Tricuspid Valve Regurgitation With the TriCinch System; NCT02098200): an observational study that aims to include 24 patients to assess the safety and performance of the device in the treatment of functional TR. The St. Antonius Hospital participates in this study.

### Edwards FORMA repair system

**Fig. 3:**
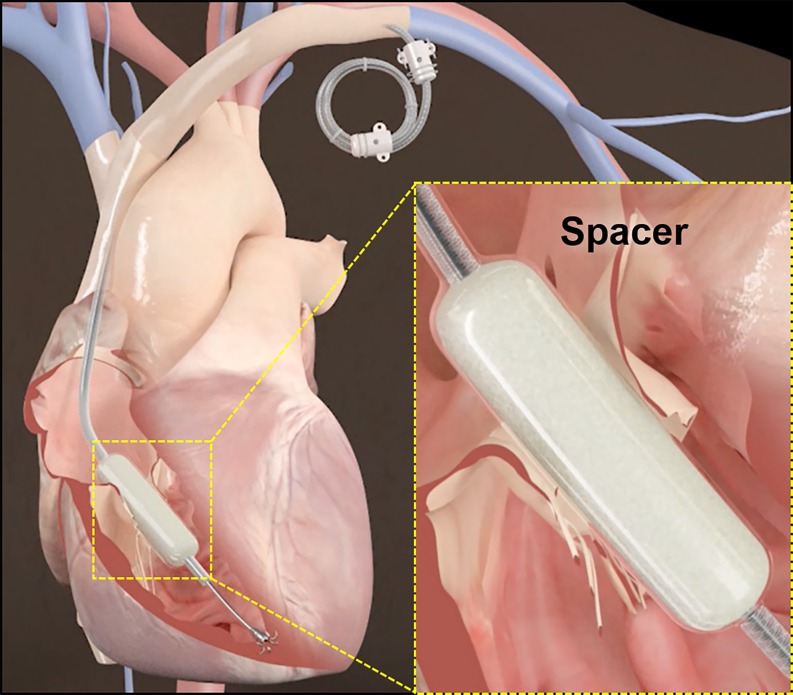
FORMA Repair System. The Forma Repair System (Edwards Lifesciences, Irvine, CA, USA) positioned at the level of the tricuspid valve annulus, with anchoring system at the right ventricular apex and excess device length coiled into a subcutaneous pocket. (Image adapted with permission from Edwards Lifesciences, Irvine, CA, USA)

The Forma Repair System (Edwards Lifesciences, Irvine, CA, USA) is a transcatheter system designed to reduce TR by occupying the regurgitant orifice area with a foam-filled polymer balloon, providing a surface for native leaflet coaptation (Fig. 3). In a first-in-man study, device implantation was successful without procedural complications in seven patients [23]. An Early Feasibility Study is on-going (NCT02471807) and a multicentre study is underway (NCT02787408).

### Millipede IRIS

The Millipede IRIS (Millipede Inc., Santa Rosa, CA, USA) is an adjustable, semi-rigid, complete annuloplasty ring that has been used clinically in the mitral and tricuspid position. The implant consists of a frame, anchors and a cinching mechanism. With the eight points of the cinching mechanism surrounding the implant the diameter of the frame can be customised to achieve proper valve leaflet coaptation.

Nine surgical patients have been treated with the IRIS ring, initially with a temporary placement series. A series of permanent implants reduced the valve diameter by up to 50% and the MR grade to zero in all but one patient. Recently, the data of the longest follow-up of approximately six months with echocardiography and computed tomography were presented [24]. The data demonstrated the feasibility and efficacy of the annuloplasty ring in surgical patients. The company is currently completing the delivery catheter which can deliver the IRIS ring via transfemoral-transseptal implantation. This device has been used in numerous animal studies.

### Cardioband

The Cardioband (Valtech Cardio, Or Yehuda, Israel) is the first transcatheter direct annuloplasty system designed for mitral and tricuspid repair. The Cardioband received its CE Mark for mitral valve repair in September 2015 [25]. A modified system for the treatment of tricuspid valve disease is anticipated later this year.

### Compassionate use

The MitraClip (Abbott Vascular, Abbott Park, IL, USA) is a single-size clip device (cobalt-chromium covered with polypropylene) with grippers above the arms to capture the leaflets making an Alfieri-like repair of the mitral valve. Recently, it has been implanted via transjugular access in 3 patients with severe TR. Using edge-to-edge repair in between all three tricuspid commissures resulted in improvement in the TR and HF symptoms [26]. Furthermore, successful transfemoral implantations have been reported [27, 28].

The Trialign Percutaneous Annuloplasty System (Mitralign Inc., Tewksbury, MA, USA) has been used successfully in the treatment of functional TR [29, 30]. In a transjugular venous approach, a pair of pledgeted sutures are placed through the tricuspid annulus via a pledget delivery catheter. A dedicated plication lock device is used to plicate the annulus, effectively bicuspidising the tricuspid valve. The enrolment of 15 patients for a prospective, multicentre, FDA-approved, early feasibility assessment study has been recently completed. The study aims to assess the early safety and performance of a tricuspid-dedicated Mitralign system in the SCOUT trial (Early Feasibility of the Mitralign Percutaneous Tricuspid Valve Annuloplasty System. NCT02574650). Possible advantages of the device are the following: it has a small footprint with only a minimal implant left behind; implantation is irrespective of the annular size and adjacent anatomic structures; a 2nd pair of pledgets can be implanted to optimise the result; the procedure can be repeated in the future; and future access and treatment options of the tricuspid valve are possible.

## Reduction of left atrial pressure

Approximately half of the patients with HF suffer from HFpEF [31]. However, despite the apparent normality of LV ejection fraction, symptoms and outcomes are similar to those with HFrEF [32], particularly during physical activity. Both entities are associated with an increase in LA pressure indicating impaired LV diastolic reserve [33, 34] and leading to pulmonary congestion [35]. The disproportionate rise in LA pressure is considered to provoke symptoms and contribute to an increased morbidity and mortality [36].

Percutaneous perforation, balloon dilation and stent implantation of the interatrial septum are established techniques to create or enlarge atrial communication [37, 38]. However, complications include excessive desaturation, spontaneous fenestration closure, stent occlusion or migration, difficulties in adjusting shunt size to achieve the desired haemodynamic effect and the inability to remove or close the shunt [37]. Two dedicated transcatheter devices to reduce LA pressure are currently under clinical investigation (Tab. 3).

**Table 3:** Transcatheter options for left atrial pressure reduction

Device	Status	Mechanism	Size	Access	Product design
IASD	CE mark May 2016 [39]	Permanent IAS shunt	8 mm	16 F	Nitinol frame
V-Wave Shunt	Proof-of-principle cohort study [40]	Permanent IAS unidirectional shunt	5 mm	14 F	Nitinol frame encapsulated with expanded polytetrafluoroethylene and 3 porcine pericardial leaflets
AFR	First-in-man [41]	Permanent IAS fenestration	6, 8 or 10 mm	10, 11 or 12 F	Nitinol double-disc wire mesh

*AFR* atrial flow reducer*, CE* Conformité Européene*, IAS* interatrial septum,* IASD* interatrial shunt device

### Interatrial shunt device

**Fig. 4:**
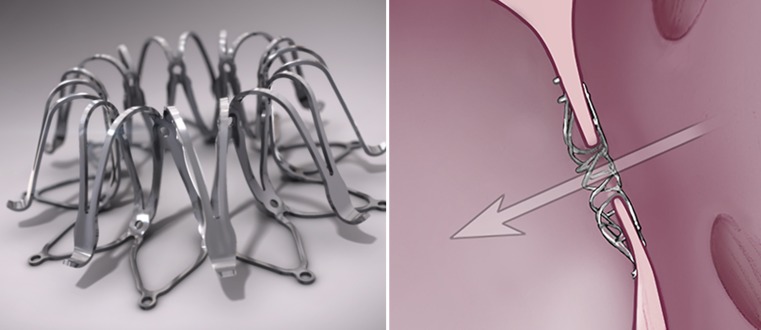
Interatrial shunt device. InterAtrial Shunt Device (IASD) system (Corvia Medical, Tewkesbury, MA, USA) (*left*) and an illustration of the final position of the device in the interatrial septum (*right*). The flow goes from the left to the right atrium. (Images adapted with permission from Corvia Medical, Tewkesbury, MA, USA)

The InterAtrial Shunt Device (IASD) system (Corvia Medical Inc., Tewkesbury, MA, USA) consists of a nitinol device (outer diameter 19 mm) inserted percutaneously in the interatrial septum to produce a permanent 8 mm atrial septal communication (Fig. 4). The design is based on predictive haemodynamic modelling which evaluated the relationship between shunt size and LA pressure reduction [39]. It is implanted after a standard transseptal puncture near the middle of the fossa ovalis (oval depression). A delivery catheter is advanced over the wire into the left atrium. Subsequently, the left side of the IASD is deployed and the delivery system is retracted to make contact with the LA side of the septum. After confirming the position, the right side of the device is deployed onto the right atrial (RA) septal side.

Results of a pilot study have been published [40, 41]. The safety and device performance was demonstrated in the REDUCE LAP-HF study (REDUCe Elevated Left Atrial Pressure in Patients with Heart Failure), in which the St. Antonius Hospital participated [42]. IASD placement was successful in 66 of 68 patients (97%). There were no major adverse events and there was no need for cardiac surgical intervention for device-related complications. Device patency was sustained. Patients had significantly fewer HF symptoms and were able to exercise longer, resulting in a substantially better quality of life. Currently, the follow-up REDUCE LAP-HF trial (NCT01913613) and a randomised REDUCE LAP-HF I trial [43] are ongoing.

### V-Wave shunt

The V‑Wave shunt device (V-Wave Ltd, [previously Akiva], Israel) is a percutaneously implanted device that creates a unidirectional shunt from the left to the right atrium. It is a self-expanding nitinol structure encapsulated with expanded polytetrafluoroethylene. The exit funnel contains three glutaraldehyde-fixed, porcine pericardial leaflets that remain in the open position and are expected to close when RA pressure exceeds LA pressure by 1–2 mmHg, preventing reverse right-to-left shunting.

A transseptal introducer sheath is advanced into the left atrium after puncturing the fossa ovalis. The device is attached with a three latches mechanism to a delivery cable, loaded into the catheter. After opening the left side in the left atrium, the system is pulled back into the interatrial septum, where the device is detached and released.

Initial safety and beneficial outcomes were reported in a single-centre proof-of-principle cohort study of 10 HFrEF patients with successful implantation [44]. At 1 month, all shunts were patent, with no thrombosis or migration. After 3 months, NYHA functional class, quality of live and 6‑minute walk distance were significantly improved. To evaluate the V‑Wave device in both HFpEF and HFrEF patients, the RELIEVE-HF trial (REducing Lung Congestion Symptoms Using thE V‑wavE Shunt in Advanced Heart Failure; NCT02511912) is planned. This is an observational study with 1‑year follow-up and estimated enrolment of 60 patients.

### Atrial flow regulator

The Atrial Flow Regulator (Mia Medical, Istanbul, Turkey) is a self-expandable double-disc wire mesh device constructed from 0.004–0.0075 inch nitinol braided into two flat discs connected by a waist of 1–2 mm and central fenestration. The device is available in 6, 8 and 10 mm fenestration diameters with a total device diameter of 18, 24 and 30 mm. The first-in-man procedure was presented in a patient with severe irreversible pulmonary arterial hypertension [45]. Besides its use in pulmonary arterial hypertension patients (right-to-left shunt), its application may well be extended to other HF populations to permit left heart decompression.

## Neuromodulators

Increased sympathetic activation and reduced parasympathetic tone, as reflected by reduced carotid baroreceptor reflex sensitivity and/or decreased heart rate variability, are potentially important contributors to HF progression associated with poor outcome. Experimentally increasing the parasympathetic tone by vagal nerve stimulation to normalise the autonomic imbalance has recently emerged as a potential therapy for HF. Several devices for vagal nerve stimulation are being developed and studied in patients with HF (Tab. 4).

**Table 4:** Transcatheter options for vagus nerve stimulation

Device	Indication	Status	Method	Location electrode
CardioFit	HFrEF	CE mark January 2009 [46]	Bidirectional (efferent/afferent) VNS	Cervical vagus approx. 3 cm below the carotid artery bifurcation and RV apex
Rheos	Persistent hypertension, HFpEF	CE mark October 2007 [47]	Afferent VNS	Carotid sinus
HASS	HFpEF + HFrEF	First-in-man	Afferent VNS	Thoracic aorta

*CE* Conformité Européene, *HFpEF* heart failure with preserved ejection fraction, *HFrEF* heart failure with reduced ejection fraction, *RV* right ventricle,* VNS* vagal nerve stimulation

### CardioFit

The CardioFit (Biocontrol Medical Ltd., Yehud, Israel) is an implantable neurostimulator system that can deliver low current adjustable electrical pulses to stimulate the vagal nerve. Parameters can be remotely programmed using a wireless system. The stimulator senses the heart rate via an intracardiac electrode and delivers stimulation at a fixed delay from the R wave. Upon implantation, the intracardiac sensing electrode is positioned at the right ventricular apex using a subclavian puncture. A cuff electrode is implanted on the cervical vagus below the carotid artery bifurcation and a stimulation lead is tunnelled under the skin to join the sensing electrode and the stimulator. An open-label study with 32 HF patients showed that chronic vagal nerve stimulation may be safe and tolerable and may improve quality of life and LV function at 1 year [46]. More recently, the randomised NECTAR-HF (NEural Cardiac TheARpy for HF) trial failed to demonstrate a significant effect on cardiac remodelling and functional capacity, but improved quality of life [47].

### Rheos system

The Rheos device (CVRx Inc., Minneapolis, MN, USA) is a carotid baroreceptor reflex stimulator intended for the treatment of resistant hypertension and HF. The non-randomised feasibility DEBuT-HT (Device Based Therapy in Hypertension) trial showed a sustained reduction of blood pressure in 17 resistant hypertensive subjects and improved functional capacity [48]. A sub-study showed a significant decrease of LV mass index and an increase in LV ejection fraction (65% to 67%) [49]. This may provide an attractive strategy for the treatment of HFpEF. The ongoing trial Rheos HOPE4HF (Health Outcomes Prospective Evaluation for Heart Failure With Ejection Fraction (EF) ≥40%, NCT01720160) will provide information in this group of patients.

### HASS system

The Harmony Aortic Stimulation System (Enopace Biomedical Ltd., Caesarea, Israel) is a minimally invasive implantable neurostimulator system capable of delivering stimulation to the aortic wall. An increase in pressure suppresses the sympathetic tone of the heart and vasculature and increases the parasympathetic tone of the heart. Impulses from the Harmony system are sent to the brain through neural pathways and result in a reduced arterial stiffness and a reduced heart rate leading to a lowered myocardial oxygen consumption and LV afterload. The stimulation parameters can be remotely programmed using a dedicated wireless communication system. Currently, the ENDO-HF (Endovascular NeuromoDulation Treatment fOr Heart Failure Patients; NCT02633644) feasibility Study, in which the St. Antonius Hospital will be participating, is recruiting to evaluate the safety and performance of the HASS device in the treatment of 20 HF subjects during a 5-year follow-up.

## Future

Newer transcatheter structural heart interventions for chronic HF are often based on surgical techniques. Some of these surgical techniques have been abandoned due to a low procedural success rate or unpredictable results. For example, surgical LV remodelling, such as the Batista procedure, in patients with a dilated cardiomyopathy was inferred not a predictable reliable alternative to transplantation [50]. Consequently, device success of the newer transcatheter devices should be carefully monitored. However, these devices could be used in patients who are not eligible for surgery due to their high operative risk. We learned from the transcatheter aortic valve implantation experience that this population might well benefit from transcatheter intervention with regard to the survival rate and quality of life. Furthermore, some of these devices might be considered as a bridge to heart transplantation rather than definite treatment, or could simply be used to improve quality of life. However, we must realise that some of these devices might even disqualify a patient for future left ventricular assist device therapy or at least complicate the implantation.

## Conclusions

Several new transcatheter structural heart interventions for chronic HF, aiming at a variety of pathophysiologic approaches, are currently being developed. Preliminary results associated with most of these new interventions are promising, with significant improvements in symptoms, functional status, quality of life and haemodynamic performance. However, the devices covered in this review are in relatively early stages of development and it is too early to compare devices within the same group based on clinical results. Data from most of these technologies are therefore restricted to first-in-man cases and observational studies, limited by experience and number of patients. We need larger randomised studies that can provide definite data on the efficacy of these devices.
